# Challenging Biopsy of a Benign Breast Lesion With Malignancy Features: Cystic Apocrine Metaplasia

**DOI:** 10.7759/cureus.20461

**Published:** 2021-12-16

**Authors:** Ahmad Kharsa, Flavia E Posleman Monetto, Quan D Nguyen

**Affiliations:** 1 Radiology, University of Texas Medical Branch, Houston, USA; 2 Radiology, University of Texas Medical Branch, Galveston, USA; 3 Radiology, Baylor College of Medicine, Houston, USA

**Keywords:** suspicious, masses, cystic, apocrine metaplasia, breast

## Abstract

Apocrine metaplasia is a benign epithelial change that primarily occurs in the terminal lobule, where the normal cuboidal epithelium is replaced by secretory apocrine cells with abundant eosinophilic cytoplasm. Even with the most recent advances in imaging modalities, radiographic findings can sometimes be equivocal in the characterization of breast lesions, leading to the necessity of tissue sampling. We report a challenging case of biopsy-proven cystic apocrine metaplasia that presented in the posterior depth with initially suspicious imaging findings concerning for malignancy. Understanding the histological basis of apocrine metaplasia and correlating it with recognized imaging features may increase diagnostic accuracy and reduce tissue resampling due to discordant histopathological results.

## Introduction

Apocrine metaplasia is a benign epithelial change that primarily occurs in the terminal lobule, where the normal cuboidal epithelium is replaced by secretory apocrine cells with abundant eosinophilic cytoplasm [1]. Apocrine metaplasia increases in incidence with age, especially in the fourth and fifth decades of life, and reaches an estimated prevalence of 50% of breasts at autopsy [2,3]. Furthermore, it commonly occurs in conjunction with other benign breast lesions, including fibrocystic changes, fibroadenoma, hamartoma, and papilloma [3]. While apocrine metaplasia in itself is not considered premalignant, atypical apocrine metaplasia may be a marker for slightly increased risk for future development of breast cancer in either breast [4,5]. Herein, we report a challenging case of biopsy-proven cystic apocrine metaplasia that presented in the posterior depth with initially suspicious imaging findings concerning for malignancy. Furthermore, we highlight the role of second-look ultrasound as a feasible method in the evaluation of breast lesions detected on magnetic resonance imaging (MRI).

## Case presentation

**Figure 1:**
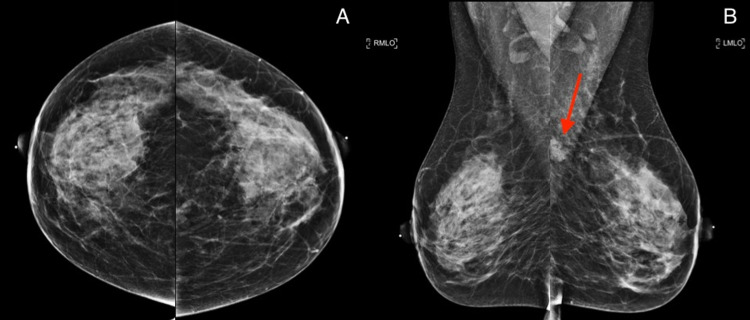
Surveillance mammograms for an asymptomatic 49-year-old patient. Craniocaudal (A) and mediolateral oblique views (B) of the bilateral breasts. There are three similar equal density masses with obscured margins seen in the upper outer quadrant of the left breast in the posterior depth on the mediolateral oblique view (red arrow). There is no evidence of suspicious masses, calcifications, or other abnormal findings in the right breast.

A 49-year-old woman presented to the imaging clinic for her annual breast imaging surveillance. She denied any history of recent breast trauma, new breast lumps, breast discharge, or weight changes. On physical examination, no dominant masses were appreciated in either breast bilaterally. Surveillance mammography revealed similar equal density masses with obscured margins and a potentially enlarged lymph node in the upper outer left breast (Figure 1).

**Figure 2:**
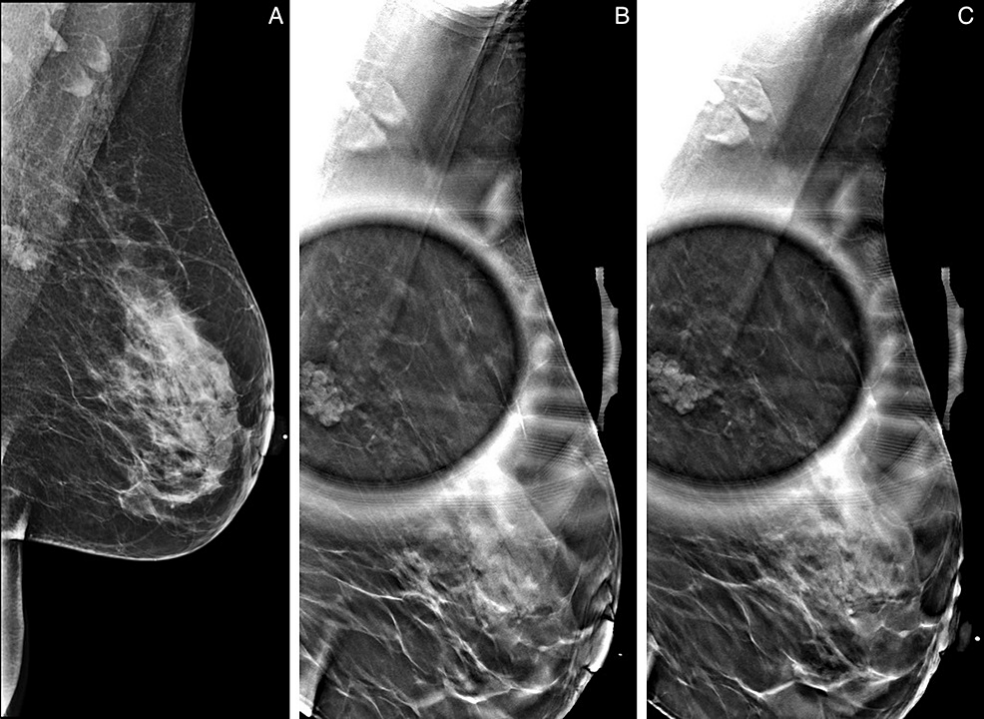
Follow-up mammograms of the left breast at six months. Mediolateral oblique view of the left breast (A) with spot magnification (B) and left axillary tail spot compression view image (C). In the retroareolar plane of the left breast in posterior depth and 11 cm from the nipple, there is a microlobulated mass measuring 18 × 10 mm.

Subsequently, follow-up sonography of the upper left breast revealed multiple anechoic lesions, ranging from 3 to 5 mm with no definite worrisome solid masses. No evidence of suspicious masses, abnormal enhancement, or other abnormal findings was detected in the right breast. On follow-up mammography at six months, the left breast lesions were noted to be larger in size (Figure 2), and ultrasound-guided biopsy was recommended.

**Figure 3:**
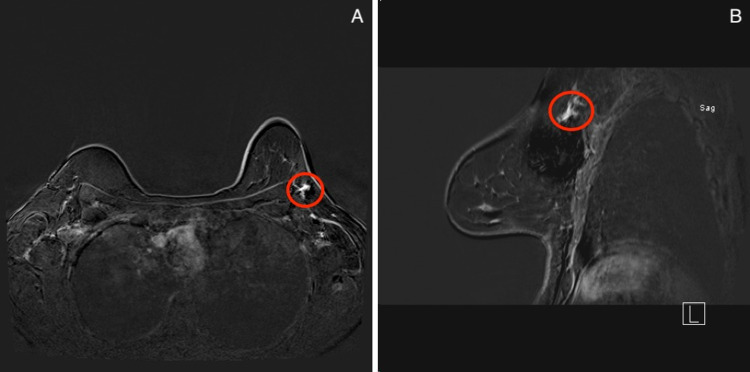
MRI of the left breast. Axial (A) and sagittal (B) T1-weighted maximum-intensity-projection contrast-enhanced with subtraction image shows an irregular shaped mass with heterogeneous enhancement, measuring 18 × 17 × 12 mm in the upper outer aspect of the left breast at the 1:00 position and 13 cm from the nipple (red circles). MRI: magnetic resonance imaging.

Nevertheless, no initial sonographic correlate was identified and further investigation with MRI was performed (Figure 3). Correlating with mammographic findings on MRI, an irregularly shaped mass in the upper outer aspect of the left breast demonstrated heterogeneous enhancement on postcontrast imaging with a suspicious washout kinetic curve pattern.

**Figure 4:**
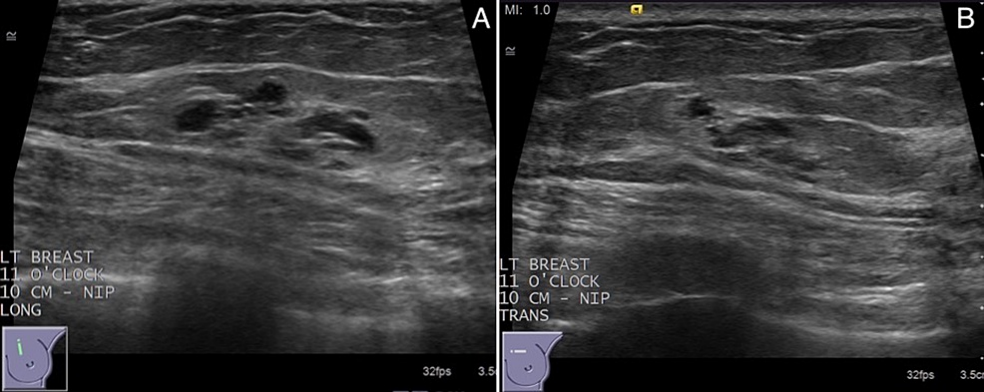
Targeted second look ultrasound of the left breast for biopsy-proven cystic apocrine metaplasia. Longitudinal (A) and transverse (B) planes on sonography demonstrate a 20 × 13 × 7 mm cluster of microcysts at the 11 o'clock position and 10 cm from the nipple.

Subsequently, a second look ultrasound was successful in the visualization of the lesion, demonstrating a 20 × 13 × 7 mm cluster of microcysts at the 11 o'clock position and 10 cm from the nipple (Figure 4). Given the concerning findings on MRI, an ultrasound-guided core needle biopsy with clip placement was performed. Histopathology confirmed the sampled tissue to be of benign breast tissue with cystic apocrine metaplasia and stromal fibrosis. Follow-up MRI imaging revealed the previously noted findings to be artifactual as no enhancement could be identified.

## Discussion

While the pathogenesis of cystic apocrine metaplasia remains to be fully elucidated, the lobular unfolding theory suggests apocrine metaplasia to be the progenitor of cyst formation. Starting in apocrine metaplastic lobules, it is believed that adjacent acini begin to unfold due to increased intraluminal pressure from secretions, forming apocrine-lined microcysts. Gradually, these microcysts continue to enlarge and fuse, forming a large unilocular cyst [4,6]. Other theories suggest that cyst formation occurs in response to fibrosis and duct obstruction, with apocrine metaplasia occurring secondarily in response to increased pressure or inflammation within the terminal duct lobular unit [6].

On mammography, the lesion usually presents as an incidental or enlarging macro-lobular, equal-density mass with smooth margins. Despite this, there are no distinguishing mammographic features of apocrine metaplasia that suggest a lesion to be purely followed by imaging. In addition, and the absence of the cytologic or histopathologic evaluation, cysts with a normal epithelium cannot be usually distinguished from apocrine metaplasia [7,8].

Similarly, the most typical sonographic appearance of apocrine metaplasia is a lobulated mass composed of small clustered anechoic foci with intervening septations and partial posterior acoustic enhancement. These anechoic foci correlate with the dilating acini histologically and become more recognizable on sonography as they fuse and increase in size [4]. Other sonographic findings include complex microcysts and microcysts with calcifications. If a solid component is identified within such cystic structures, a biopsy is warranted as concurrent malignant processes may be present [4,7].

When the mammographic or sonographic findings are indeterminate in the characterization of breast lesions, MRI has become the tool of choice in elucidating such lesions and effectively screening for malignancy. Several MRI features have been associated with apocrine metaplasia including T2-hyperintense lesions, sub-centimeter lesions with smooth margins, and hybrid lesions with mass and non-mass features. Some propose that the spectrum of mass and non-mass features is due to minimal dilation of the lobular unit in the early stage, such as the enhanced epithelium appearing as a linear non-mass lesion. As the lobular unit dilates, the lesion takes on a mass appearance with enhancing apocrine epithelium [6,8]. In addition, as was our case, the presence of washout kinetics does not preclude benign apocrine metaplasia from the differential diagnosis. In fact, in a recent MRI review, 58% of apocrine metaplasia lesions were associated with washout kinetics [6].

As it can be seen, even with the most recent advances in imaging modalities, radiographic findings can sometimes be equivocal in the characterization of breast lesions, leading to the necessity of tissue sampling. In our case, given the difficult location of the lesion in the posterior depth and the lack of an initial sonographic correlate, the biopsy was initially planned to be performed under MRI guidance. Nevertheless, MRI-guided biopsy has several limitations, including lengthy procedure time and high cost, required intravenous administration of contrast, and difficult access to posterior lesions [6]. As such, as a last effort, a second-look ultrasound was performed and was shown to be successful to characterize the breast lesions and guide tissue sampling. Ultimately, it is to be noted that malignant lesions may present with benign findings on second-look ultrasound and a lower threshold should be utilized in such cases to consider biopsy or excision.

## Conclusions

Apocrine metaplasia is a common yet benign epithelial alteration with radiographic findings that can sometimes mimic malignant processes. Understanding the histological basis of apocrine metaplasia and correlating it with clinical history and recognized imaging features may increase diagnostic accuracy and reduce tissue resampling due to discordant histopathological results. Nevertheless, in equivocal and challenging cases, an image-guided biopsy is needed to definitively rule out underlying malignant processes.
